# To the editor

**DOI:** 10.1007/s10877-014-9644-9

**Published:** 2015-01-21

**Authors:** Scott Kelley, Michal Ronen, Joshua Colman, Rachel Weissbrod

**Affiliations:** 1 Medical Affairs, Covidien, Mansfield, MA USA; 2 Research & Development, Covidien, Jerusalem, Israel

Tanaka et al. [1] concluded that acoustic monitoring had a higher probability for detecting and a lower probability for missing respiratory pauses of 20-s duration than capnography. However, we believe that critical issues related to methods used for capnography data collection and analysis warrant careful assessment of their conclusion that acoustic monitoring performs better than capnography.

This pilot study in a convenience sample of 20 patients during procedural sedation yielded only 51 episodes of confirmed respiratory pauses for comparative analysis of the different detection methods. Of note, the authors selected a duration of 20-s to indicate a “respiratory pause” event, although in other studies in the perioperative and sedation setting, a 30-s duration has been used to define clinically significant episodes of respiratory pause. Tanaka et al. could have described the duration of the 51 respiratory pause events they recorded to better characterize the types of events in their patients.

The most important concern relates to the potential influence of capnography collection and analysis on the estimates of capnography performance. As described, all EtCO_2_ monitoring samples were collected from tubing connected to a standard nasal cannula. An oral-nasal sampling cannula, unlike a nasal-only cannula, enables improved capture of the patient’s breath during ‘mouth breathing’ episodes commonly observed during sedation [2, 7]. This type of breath sampling bias is a reasonable and likely explanation for many of the 62 “false positive” episodes of respiratory pauses associated with capnography. In addition, the authors used two capnography systems for “standard of care” monitoring and for data analysis. Use of a single sampling line connected to two capnography devices could potentially decrease monitor performance. For the Capnostream 20p device utilized for data analysis, the manufacturer’s instructions for use specify the device be used with a Microstream^®^ sampling line. In short, the capnography methods used to collect and analyze patient breaths likely created multiple “false positive” events because oral breaths were not sampled and capnography analysis was compromised.

The approach used to report performance of the different methods to detect respiratory pauses does not follow current approaches in the areas of respiratory monitoring devices. For example, the Food and Drug Administration provides guidance regarding validating the performance of apnea monitoring devices.^1^ Because of the ill-defined nature of true negatives during continuous monitoring, the FDA discourages reporting of specificity values for apnea monitors. The suggested diagnostic measures to report include sensitivity, positive predictive value and false alarm rates and percentages. The positive predictive value (PPV = (TP/(TP + FP))(100 %)) helps provide the user with a better indication of how often an alarm represents true apnea. The overemphasis of the specificity calculation to measure the performance of the different detection methods is inappropriate and misleading. The data demonstrate that capnography was far more sensitive in detecting respiratory pauses than the acoustics technology (88 vs 55 %). The decreased PPV for capnography in this study (42 %) is influenced by the large number of false positive observations related to methodological issues discussed above.

In summary, the methods and subsequent analysis of capnography data utilized in this study markedly limit the potential conclusions as stated by the author. The high sensitivity of capnography to detect respiratory events, and the improved specificity provided with better sampling devices have contributed to the positive results and clinical impact of capnography monitoring during procedural sedation [3–7]. Alternative approaches, such as acoustic monitoring, need similarly designed and sized studies to establish their clinical effectiveness.

## Footnotes

1
http://www.fda.gov/MedicalDevices/DeviceRegulationandGuidance/GuidanceDocuments/ucm072846.htm (Accessed August 7, 2014).
